# *Microbispora clausenae* sp. nov., an endophytic actinobacterium isolated from the surface-sterilized stem of a Thai medicinal plant, *Clausena excavala* Burm. f.

**DOI:** 10.1099/ijsem.0.004518

**Published:** 2020-10-23

**Authors:** Onuma Kaewkla, Wilaiwan Koomsiri, Arinthip Thamchaipenet, Christopher Milton Mathew Franco

**Affiliations:** ^1^​ Department of Biology, Faculty of Science, Mahasarakham University, Maha Sarakham 44150, Thailand; ^2^​ Department of Medical Biotechnology, College of Medicine and Public Health, Flinders University, Bedford Park, SA 5042, Australia; ^3^​ Department of Genetics, Kasetsart University, Chatuchuk, Bangkok 10900, Thailand; ^4^​ Omics Center for Agriculture, Bioresources, Food and Health, Kasetsart University (OmiKU), Bangkok 10900, Thailand; ^5^​ Department of Genetics, Kasetsart University, Chatuchak, Bangkok 10900, Thailand

**Keywords:** endophyte, genome, *Microbispora clausenae*, polyphasic taxonomy

## Abstract

An endophytic actinobacterium, strain CLES2^T^, was discovered from the surface-sterilized stem of a Thai medicinal plant, *Clausena excavala* Burm. f., collected from the Phujong-Nayoa National Park, Ubon Ratchathani Province, Thailand. The results of a polyphasic taxonomic study identified this strain as a member of the genus *Microbispora* and a Gram-stain-positive, aerobic actinobacterium. It had well-developed substrate mycelia, which were non-motile and possessed paired spores. A phylogenetic evaluation based on 16S rRNA gene sequence analysis placed this strain in the family *Streptosporangiaceae *, being most closely related to *Microbispora bryophytorum* NEAU-TX2-2^T^ (99.4 %), *Microbispora camponoti* 2C-HV3^T^ (99.2 %), *Microbispora catharanthi* CR1-09^T^ (99.2 %) and *Microbispora amethystogenes* JCM 3021^T^ and *Microbispora fusca* NEAU-HEGS1-5^T^ (both at 99.1 %). The major cellular fatty acid of this strain was iso-C_16 : 0_ and major menaquinone was MK-9(H_4_). The polar lipid profile of strain CLES2^T^ contained diphosphatidylglycerol, phosphatidylmethylethanolamine, phosphatidylinositol and phosphatidylinositol dimannosides. These chemotaxonomic data confirmed the affiliation of strain CLES2^T^ to the genus *Microbispora *. The DNA G+C content of this strain was 70 mol%. Digital DNA–DNA hybridization and average nucleotide identity blast values between strain CLES2^T^ and *M. catharanthi* CR1-09^T^ were 62.4 and 94.0 %, respectively. The results of the polyphasic study allowed the genotypic and phenotypic differentiation of strain CLES2^T^ from its closest species with valid names. The name proposed for the new species is *Microbispora clausenae* sp. nov. The type strain is CLES2^T^ (=DSM 101759^T^=NRRL B-65340^T^).

The genus *Microbispora* belongs to the family *Streptosporangiaceae* [1]. This genus contains *meso*-diaminopimelic acid in the cell-wall peptidoglycan. The phospholipid is type IV, which contains phosphatidylcholine and unknown glucosamine-containing compounds, but no phosphatidylglycerol. Predominant menaquinones have nine isoprene units: MK-9(H_2_), MK-9(H_4_), MK-9(H_0_) and small amount of MK-9(H_6_). The fatty acid profile is a complex mixture of saturated, unsaturated, iso-, anteiso- and branched-chain fatty acids [2]. The genus *Microbispora* contains 12 species, which were discovered from various habitats such as soil, plant tissues and insects. Six species were isolated from soil, namely *Microbispora rosea* subsp. *rosea *, *Microbispora rosea* subsp. *aerata* [3]*, Microbispora coralline* [4], *Microbispora siamensis* [5], *Microbispora hainanensis* [6] and *Microbispora soli* isolated from hot spring soil [7]. Five species were reported as endophytes which were isolated from different types of plant tissues namely *Microbispora bryophytorum* from moss [8], *Microbispora catharanthi* from *Catharanthus roseus* [9] and *Microbispora triticiradicis *, *Microbispora tritici* and *Microbispora fusca* from wheat [10–12]. One species, *Microbispora camponoti *, was associated with a Japanese carpenter ant (*Camponotus japonicas*) [13].

Strain CLES2^T^ was isolated from the stem sample of a Thai medicinal plant (*Clausena excavala* Burm. f.) collected from the Phujong-Nayoa National Park, Ubon Ratchathani Province, Thailand (14.438954° N 105.344589° E), and processed within 4 h of collection [14]. Surface-sterilized stem tissue was placed onto VL70 medium containing a defined amino acid mixture and solidified with 0.8 % gellan gum [14, 15]. Strain CLES2^T^ emerged as a small colony from the stem tissue after incubation for 2 weeks at 27 °C. Polyphasic taxonomy showed that this strain represents a novel species of the genus *Microbispora *, for which the name *Microbispora clausenae* sp. nov. is proposed.

Genomic DNA of strain CLES2^T^ was extracted and used for 16S rRNA gene amplification and sequencing as described previously [14]. The 16S rRNA gene sequence of CLES2^T^ was analysed using the EzTaxon-e server (www.ezbiocloud.net) [16]. The 16S rRNA gene sequences of representatives of all valid strains of the genus *Microbispora* available from GenBank/EMBL were subsequently aligned with strain CLES2^T^ using clustal_x [17] with *Nonomuraea cavernae* SYSU K10005^T^ as the outgroup. The phylogenetic trees were reconstructed based on the maximum-likelihood and neighbour-joining algorithms using the software package mega version X [18]. The Tamura–Nei model [19] was applied to the maximum-likelihood analysis using the Subtree-Pruning-Regrafting-Extensive (SPR level 5) program. The neighbour-joining algorithm [20] was used according to Kimura’s two-parameter model [21]. The topology of the tree was evaluated by performing a bootstrap analysis [22] based on 1000 replications.

The phylogenetic trees clearly revealed that strain CLES2^T^ was a member of the genus *Microbispora* (Figs 1 and S1, available in the online version of this article).

**Fig. 1.**
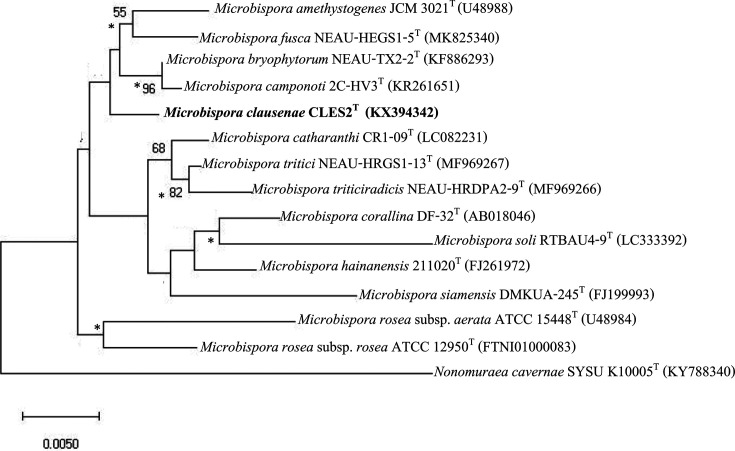
16S rRNA gene-based neighbour-joining tree showing the phylogenetic relationships between *Microbispora clausenae* CLES2^T^ and related strains with valid names belonging to the genus *Microbispora *. *Nonomuraea cavernae* SYSU K10005^T^ was used as an outgroup. Sequence length was 1423 bp. Bootstrap values (>50 %) based on 1000 replicates are shown at the branch nodes and asterisks (*) indicate clades that were conserved in the maximum-likelihood and neighbour-joining trees. The scale bar represents 0.005 changes per nucleotide.

The 16S rRNA gene similarities of strain CLES2^T^ (1452 nt) to its related species were 99.4 % to *M. bryophytorum* NEAU-TX2-2^T^ *,* 99.2 % to both *M. camponoti* 2C-HV3^T^ and *M. catharanthi* CR1-09^T^ and 99.1 % to both *M. amethystogenes* JCM 3021^T^ and *M. fusca* NEAU-HEGS1-5^T^.

The results showed that strain CLES2^T^ formed a different cluster with these closest type strains. The closest neigbours on both phylogenetic trees were *M. bryophytorum* NEAU-TX2-2^T^ and *M. camponoti* 2C-HV3^T^, which shared 16S rRNA gene similarity at 99.4 and 99.2 %, respectively. Other closest neighbours were *M. amethystogenes* JCM 3021^T^ and *M. fusca* NEAU-HEGS1-5^T^. The type strain, *M. catharanthi* CR1-09^T^, which had 99.2 % 16S rRNA gene similarity to and had the highest values of ANIb, ANIm and dDDH with strain CLES2^T^ was located at the farthest distance when compared with the other four related species (Figs 1 and S1). According to 16S rRNA gene similarity and position of strain CLES2^T^ on the phylogenetic trees, the four type strains *M. bryophytorum* NEAU-TX2-2^T^, *M. catharanthi* CR1-09^T^, *M. camponoti* 2C-HV3^T^ and *M. amethystogenes* JCM 3021^T^ were selected for comparative physiological and biochemical studies.

Genomic DNA for whole genome sequencing of strain CLES2^T^ was extracted using GenElute (Sigma) and a short insert size library was prepared. The genome was sequenced by the Hiseq X-ten platform (Illumina; 2×150 bp paired-end reads) at the Beijing Genome Institute (BGI; Hong Kong). *De novo* assembly of the reads was achieved by using Unicycler (version 0.4.8; without long reads) [23].

The draft assembly of the genome of strain CLES2^T^ was submitted to GenBank with the accession number JACBWX000000000. The phylogenetic tree of the genomes of strain CLES2^T^ and its related taxa was reconstructed using the Type (strain) Genome Server (TYGS) [24, 25]. The tree inferred with FastME version 2.1.6.1 [26] from genome blast distance phylogeny (GBDP) and distances were calculated from genome sequences. The branch lengths were scaled in terms of GBDP distance formula *d4*.

The average nucleotide identity (ANI) values between strain CLES2^T^ and four related species were evaluated with pairwise genome alignment by using the ANI-blast (ANIb) and ANI-MUMmer (ANIm) algorithms [27]. Correlation indexes of tetra-nucleotide signature (Tetra) were applied within the JSpecies Web Server [27, 28]. Digital DNA–DNA hybridization (dDDH) values were calculated by applying the Genome-to-Genome Distance calculator (GGDC 2.1; blast+ method) in which formula 2 (identities/HSP length) was applied to the incomplete draft genome [24].

The draft genome sequence of strain CLES2^T^ was 7.25 Mb with a DNA G+C content of 70 mol%. The genome analysis resulted in the following ANIb and ANIm values between the draft genome of strain CLES2^T^ and its related species: *M. catharanthi* CR1-09^T^(94.0 and 95.6 %), *M. bryophytorum* NEAU-TX2-2^T^(92.0 and 94.1 %) and *M. fusca* NBRC 13915^T^(86.0 and 89.8 %). According to the report of Richter and Rosselló-Móra [28], the ANI species delineation cutoff is 95–96 %. However, the ANIm value between strain CLES2^T^ and *M. catharanthi* CR1-09^T^ was 95.6 % (Table 1). An investigation by Kim *et al*. [29] revealed that some strains were identified as novel species having an ANI value higher than 96 %. Therefore, the differential characteristics between strain CLES2^T^ and this type strain should be considered thoroughly. The Tetra values between strain CLES2^T^ and *M. catharanthi* CR1-09^T^, *M. bryophytorum* NEAU-TX2-2^T^ and *M. fusca* NBRC 13915^T^ were 0.9982, 0.9980 and 0.9942, respectively, which were well below the cut off value of ≥0.999 for the same species [28].

**Table 1.** Average nucleotide identity, digital DNA–DNA hybridization and Tetra values between strain CLES2^T^ and its related species Strain: 1, CLES2^T^; 2, *Microbispora bryophytorum* NEAU-TX2-2^T^; 3, *Microbispora catharanthi* CR1-09^T^; 4, *Microbispora fusca* NBRC 13915^T^; 5*, Microbispora hainanensis* DSM 45428^T^; 6, *Microbispora rosea* ATCC 12950^T^; 7, *Microbispora triticiradicis* NEAU-HRDPA2-9^T^.

Strain/ Analysis	1	2	3	4	5	6	7
ANIb (%)	–	92.0	94.0	86.0	90.9	91.7	86.0
ANIm (%)	–	94.1	**95.6**	89.8	92.9	93.7	89.8
dDDH (%)	–	54.1 (C.I. model 51.4–56.8)	62.4 (C.I. model 59.5–65.2)	36 (C.I. model 33.6–38.5)	48 (C.I. model 45.4–50.6)	51.6 (C.I. model 48.9–54.2)	36 (C.I. model 33.5–38.5)
Tetra (Z score)	–	0.9982	0.9980	0.9942	0.9964	0.998	0.994

The dDDH values between the genome of strain CLES2^T^ and those of three related species, *M. catharanthi* CR1-09^T^, *M. bryophytorum* NEAU-TX2-2^T^ and *M. fusca* NBRC 13915^T^, were 62.4, 54.1 and 36 %, respectively (Table 1). These values are lower than the threshold of 70 % used to define species [25, 30].

The phylogenetic tree based on the TYGS revealed the relationship between strain CLES2^T^ and the related type strains (Fig. 2). The result clearly showed that strain CLES2^T^ was positioned in a different node with its closest strains, *M. bryophytorum* NEAU-TX2-2^T^ and *M. catharanthi* CR1-09^T^. Also, the phylogenetic tree of the genome showed that strain CLES2^T^ was placed in a different species cluster from these two type strains (Fig. 2).

**Fig. 2.**
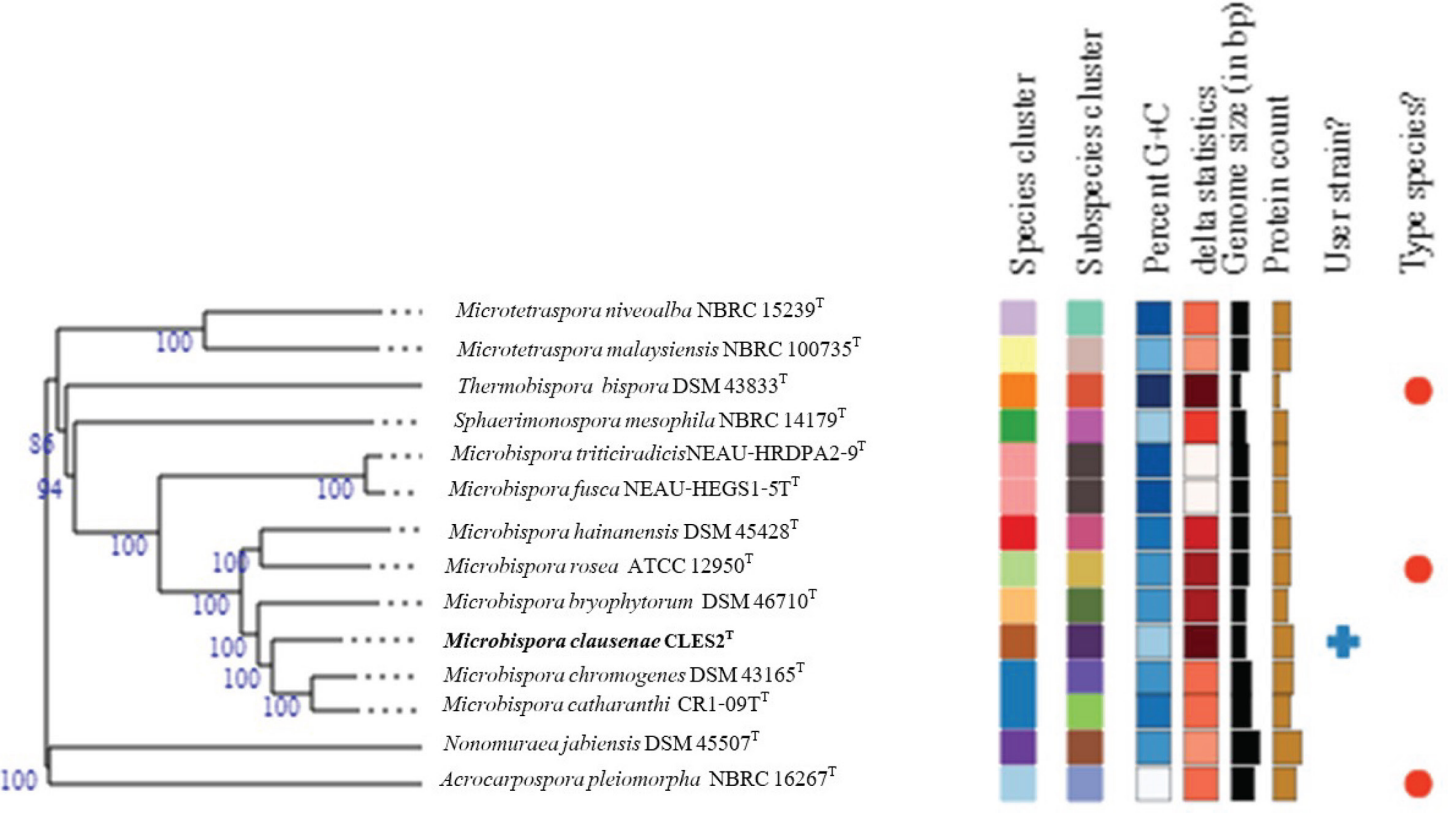
Phylogenomic tree based on TYGS results showing the relationship between strain CLES2^T^ and its related type strains. The numbers above branches are GBDP pseudo-bootstrap support values >60 % from 100 replications, with an average branch support of 98.2 %. The tree was rooted at the midpoint [43]. Leaf labels are annotated by affiliation to species and subspecies clusters, genomic G+C content, δ values and overall genome sequence length, number of proteins, and the kind of strain [24].

Whole-cell sugar was analysed by the TLC method of Hasegawa *et al.* [31] and diaminopimelic acid (DAP) was identified by TLC using the method of Bousfield *et al.* [32]. The *meso*-isomer of DAP was detected from strain CLES2^T^ and the whole-cell sugar contained galactose, glucose, mannose and madurose, while the whole-cell sugars of the closest type strains, *M. bryophytorum* NEAU-TX2-2^T^, were glucose and madurose [8] and *M. catharanthi* CR1-09^T^ contained galactose, glucose, madurose and a small amount of xylose [9].

The phospholipid pattern was determined as described by Minnikin *et al.* [33] and Komagata and Suzuki [34] using 5 % ethanolic molybdophospholric acid, ninhydrin, molybdenum blue reagent, α-naphthol, periodate-Schiff spray and Dragendorff reagent. The major lipids were diphosphatidylglycerol, phosphatidylmonomethylethanolamine, phosphatidylethanolamine, phosphatidylinositol, phosphatidylinositol dimannosides and four unknown and unidentified lipids that tested positive with ninhydrin and molybdenum blue reagents (Fig S2), which corresponds to phospholipid type IV [35].

Isoprenoid quinones were extracted and purified using the method of Minnikin *et al.* [34] and analysed by reverse phase LC-MS employing UV detection and electrospray mass spectrometry (ESI) according to Kaewkla and Franco [36].

Strain CLES2^T^ contained MK-9(H_2_) (43.4 %) as the predominant menaquinone and MK-9(H_4_) (33.8 %) and MK-9(H_0_) (23.1 %), while *M. catharanthi* CR1-09^T^ contained MK-9(H_4_) (50 %), MK9(H_2_) (34 %), MK-9(H_0_) (11 %) and MK-9(H_6_) (5 %) [9] – the latter menaquinone was not detected in strain CLES2^T^.

For the analysis of whole-cell fatty acids, strain CLES2^T^ and its three closest type strains were grown for 7 days at 27 °C in tryptic soya broth (Oxoid) in an Erlenmeyer flask at 150 r.p.m. and harvested by centrifugation. Washed cells (100 mg) were saponified, methylated and extracted, and then the fatty acid methyl esters (FAMEs) were determined by following the protocols described by Microbial Identification Inc. (midi) [37]. The sactin6 method and Sherlock version 6.3 were used for analysis.

The whole-cell fatty acid pattern of strain CLES2^T^ was of the *iso-*branched type (Table 2). The major cellular fatty acids of this strain were iso-C_16 : 0_ (43.3 %), C_17 : 0_ 10-methyl (18 %) and iso-C_15 : 0_ (12.4 %), which was the same pattern found in all related type strains including *M. catharanthi* CR1-09^T^ [9].

**Table 2.** Cellular fatty acids (%) of strain CLES2^T^ and related species of *Microbispora
* Strain: 1, CLES2^T^; 2, *Microbispora bryophytorum* NEAU-TX2-2^T^; 3, *Microbispora camponoti* 2C-HV3^T^; 4, *Microbispora amethystogenes* JCM 3021^T^. Only fatty acids detected at more than 0.5 % of the total are presented. –, Not detected. All the data are from this study.

Fatty acid	1	2	3	4
iso-C_14 : 0_	1.2	2.5	2.6	2.4
C_14 : 0_	0.6	1.4	–	–
iso-C_15 : 0_	**12.4**	**15.6**	**18.1**	**7.9**
anteiso-C_15 : 0_	–	2.0	5.9	1.3
C_15 : 0_	5.4	4.6	3.8	9.5
iso-H-C_16 : 1_	0.7	–	–	–
iso-C_16 : 0_	**43.3**	**32.9**	**35.3**	**39.1**
C_16 : 1_ *cis*9	0.9	1.8	–	0.8
C_16 : 0_	1.6	3.1	1.8	3.6
C_16 : 0_ 10-methyl	3.3	6.2	2.2	2.3
iso-C_17 : 0_	2.4	3.8	5.0	2.9
anteiso-C_17 : 0_	0.6	2.0	1.8	2.1
C_17 : 1_ *cis*9	0.8	1.3	0.8	2.3
iso-C_16 : 0_ 2OH	2.7	0.6	0.6	0.3
C_17 : 0_	0.9	1.1	1.7	5.3
C_17 : 0_ 10-methyl	**18.0**	**14.5**	**14.1**	**13.3**
iso-C_18 : 0_	0.7	0.6	1.3	1.3
C_18 : 1_ *cis*9	–	0.6	–	–
C_18 : 0_	1.1	1.1	1.1	0.7
iso-C_17 : 0_ 2OH	–	–	–	0.7
C_18 : 0_ 10-methyl TBSA	–	1.4	0.7	–
iso-C_17 : 1_ I	–	0.6	–	–

The results of our chemotaxonomic study showed that strain CLES2^T^ was clearly different from *M. catharanthi* CR1-09^T^.

The morphological characteristics of strain CLES2^T^ and the three closest type strains were studied on eight different media: International *Streptomyces* Project (ISP) 2, ISP 3, ISP 4, ISP 5, ISP 7 [38, 39], Bennett’s agar, half-strength potato dextrose agar and nutrient agar [39]. Colour determination was based on the *Methuen Handbook of Colour* [40]. Strain CLES2^T^ showed morphology belonging to the genus *Microbispora *, with a substrate mycelium that was well developed and an aerial mycelium formed well in some media. Cultural characteristics on different media are demonstrated in Table S1. Electron microscopy revealed that it formed paired spores (approximately 1×0.8 microns) with smooth surfaces (Fig. S3).

The physiological and biochemical characteristics of strain CLES2^T^ and its four closest type strains were studied. Acid production from 23 carbohydrates and decomposition of l-tyrosine, urea and aesculin were evaluated according to the methods of Gordon *et al.* [41]. Hydrolysis of starch, catalase production, assimilation of seven organic acids and utilization of four phenolic compounds as sole carbon source were described by Kurup and Schmitt [42]. Growth at different temperatures (4, 15, 27, 37, 45 and 55 °C), NaCl concentrations (1, 3, 5, 10, 15 and 20 %, w/v) and pH between pH 4 and 10 (in 1 pH unit intervals) were evaluated after incubation at 37 °C for 7–14 days on ISP 2 medium [42].

The physiological properties of strain CLES2^T^ and its closest neighbours, *M. bryophytorum* NEAU-TX2-2^T^ and *M. catharanthi* CR1-09^T^, were significantly different (Table 3).

**Table 3.** Differential characteristics between strain CLES2^T^ and related species of *Microbispora
* Strain: 1, CLES2^T^; 2, *Microbispora catharanthi* CR1-09^T^; 3, *Microbispora bryophytorum* NEAU-TX2-2^T^; 4, *Microbispora camponoti* 2C-HV3^T^; 5, *Microbispora amethystogenes* JCM 3021^T^; +, Positive or present; w, weakly positive; −, negative or absent; nd, not done. Catalase was positive for all strains. All strains could produce acid from arabinose, fructose, galactose, glucose, mannose, mannitol, sucrose and xylose but not from sorbitol. All strains could assimilate acetate but not tartrate. They could not use phenol and benzene as sole carbon sources. All strains could hydrolyse aesculin. They could grow at 1 % NaCl (w/v) but not at 15, and 20 % (w/v) NaCl. All strains could grow at between pH 6 and 10 and between 27 and 37 °C but could not grow at 4 and 55 °C and at pH 4.

Characteristics	1	2	3	4	5	Characteristics	1	2	3	4	5
Colour of spores on ISP2	Reddish pink	Pinkish white	White pink	White	White	Soluble pigment on ISP2	Dark brown	Dark purple	−	−	Dark brown
Acid production from:						Organic acid use:					
Cellulose	w	nd	+	+	+	Citrate	w	+	+	w	−
Cellobiose	+	+	nd	nd	nd	Lactate	+	nd	+	+	−
Ducitol	−	−	nd	nd	nd	Malate	+	−	−	−	−
Fucose	+	nd	−	−	+	Propionate	+	−	−	−	+
Maltose	+	w	−	−	+	Growth with/at:					
*Myo*-inositol	+	w	−	−	w	3% NaCl	+	+	+	w	+
Methyl d-glucopyranoside	+	nd	−	−	+	5% NaCl	−	−	+	−	w
*meso*-Erythritol	−	nd	+	−	−	10% NaCl	−	−	+	−	−
Raffinose	w	−	+	−	−	45 °C	+	+	−	−	+
Rhamnose	+	−	nd	nd	nd	pH 5	w	−	+	w	w
Ribose	+	+	+	+	w	Use of phenolic compounds:					
Salicin	+	+	nd	nd	nd	Pyridine (sigma)	+	−	−	−	−
Trehalose	+	−	+	+	+	Toluene	+	−	−	−	−
Decomposition of:											
l-Tyrosine	+	nd	−	−	+						
Starch	+	−	+	−	+						
Skimmed milk	+	−	+	+	−						
Urea	−	−	+	+	−						

Strain CLES2^T^ could produce acid from fucose, maltose, *myo*-inositol and methyl d-glucopyranoside, but the closest type strain, *M. bryophytorum* NEAU-TX2-2^T^, could not. In contrast, the closest type strain could produce acid from *meso*-erythritol but strain CLES2^T^ could not. Also, strain CLES2^T^ could decompose *l*-tyrosine, assimilate malate and propionate, grow at 45^ ^°C and use pyridine and toluene as sole carbon sources but the closest type strain could not. On the other hand, the closest type strain could decompose urea, grow at 5 and 10 % NaCl (w/v) but strain CLES2^T^ could not.

Based on the data of ANIb and ANIm including dDDH, strain CLES2^T^ shared the highest values with *M. catharanthi* CR1-09^T^. The physiology and biochemical properties of these two strains were compared. The result showed that strain CLES2^T^ differed significantly from this reference strain. Strain CLES2^T^ could not produce soluble pigment, but the reference strain could. The spore colour of strain CLES2^T^ was reddish white on ISP 2 and ISP 7, but that of the reference strain was pinkish white. In addition, strain CLES2^T^ could hydrolyse starch and skimmed milk, while the reference strain could not. Also, strain CLES2^T^ grew weakly at pH 5 and 15 °C, but the reference strain could not. They differed in terms of acid production and organic assimilation. Strain CLES2^T^ produced acid from raffinose, rhamnose and trehalose and assimilated propionate and malate, but the reference strain could not. Also, strain CLES2^T^ produced acid from *myo*-inositol and maltose, but the reference strain could only do so weakly. Also, strain CLES2^T^ could use pyridine and toluene as sole carbon sources, but the reference strain could not.

Based on the results of this polyphasic study, strain CLES2^T^ is proposed to represent a novel species of the genus *Microbispora *, named *Microbispora clausenae* sp. nov.

## Description of *Microbispora clausenae* sp. nov.

*Microbispora clausenae* (clau′se.nae. N.L. gen. n. *clausenae* of *Clausena*, pertaining to the plant from which the type strain was isolated).

Aerobic and catalase-positive. Grows between 15 and 45 °C, but best growth occurs between 27 and 45 °C. Grows well between pH 6.0 and 10.0 and in the presence of 3 % (w/v) NaCl. Colonies are wrinkled with a dry surface. Substrate mycelium develops well on most media and aerial mycelium forms well on some media. Diffusible pigments are observed on ISP 2. The mycelium is extensively branched and forms paired spores. Paired rod-shaped spores (0.8×1.0 µm) are observed. Produces acid from arabinose, cellobiose, fucose, fructose, galactose, glucose, mannose, mannitol, maltose, *myo*-inositol, methyl d-glucopyranoside, sucrose, trehalose, rhamnose, ribose, salicin, trehalose and xylose, but not from ducitol, *meso*-erythritol or sorbitol. Assimilates acetate, citrate, lactate, malate and propionate, but not tartrate. Decomposes l-tyrosine, starch and skimmed milk, but not urea. Uses pyridine and toluene, but not phenol and benzene as a sole carbon source.

It is characterized by *meso*-diaminopimelic acid in its peptidoglycan layer and galactose, glucose, mannose and madurose as whole-cell sugars. Phospholipids are diphosphatidylglycerol, phosphatidylmethylethanolamine, phosphatidylethanolamine, phosphatidylinositol, phosphatidylinositol dimannosides and four unknown glycolipids. Major cellular fatty acids are iso-C_16 : 0_, C_17 : 0_ 10-methyl and iso-C_15 : 0_. MK-9(H_2_), MK-9(H_4_) and MK-9(H_0_) are predominant menaquinones. The DNA G+C content of the type strain is 70 mol%.

The type strain, CLES2^T^ (=DSM 101759^T^=NRRL B-65340^T^), is an endophytic actinobacterium isolated from the stem of a Thai medicinal plant, *Clausena excavala* Burm. f., which grows in Phujong-Nayoa National Park, Ubon Ratchathani Province, Thailand. The GenBank/EMBL/ DDBJ accession number for the 16S rRNA gene sequence of strain CR1-09T is KX394342. The Whole Genome Shotgun project of strain CLES2^T^ has been deposited at DDBJ/ENA/GenBank under the accession JACBWX000000000. The version described in this paper is version JACBWX000000000.

## Supplementary Data

Supplementary material 1
