# Supplementary material for: Microbispora clausenae sp. nov., an endophytic actinobacterium isolated from the surface-sterilized stem of a Thai medicinal plant, Clausena excavala Burm. f
Source: Int J Syst Evol Microbiol. 2020 Oct 23;70(12):6213–9. doi: 10.1099/ijsem.0.004518 (PMC8049491; doi:10.1099/ijsem.0.004518)
Supplement: Supplementary material 1 [file ijsem-70-6213-s001.pdf]

Microbispora clausenae sp. nov., an endophytic actinobacterium isolated from the surface-sterilized stem of a Thai medicinal plant, *Clausena excavala* Burm. f.

Onuma Kaewkla[1,3], Wilaiwan Koomsiri[2], Arinthip Thamchaipinet[2], Christopher Milton Mathew Franco[3*]

[1]Department of Biology, Faculty of Science, Mahasarakham University, Maha Sarakham, 44150, Thailand

[2]Department of Genetics, Kasetsart University, Chatuchuk, Bangkok, 10900, Thailand

[3]Department of Medical Biotechnology, School of Medicine, Flinders University, Bedford Park, SA 5042, Australia

* Corresponding author:

Christopher Milton Mathew Franco

Email address: Chris.Franco@flinders.edu.au

Figure Legend:

Supplementary Fig. S1 16S rRNA gene-based maximum-likelihood algorithm showing the phylogenetic relationships between *Microbispora clausenae* CLES2^T and related strains with valid names belonging to the genus *Microbispora* with *Nonomuraea cavernae* SYSU K10005^T as the outgroup. Sequence length was 1423 bp. Bootstrap values (>50 %) based on 1000 replicates are shown at the branch nodes and asterisk (*) indicates clades that were conserved in the maximum-likelihood and neighbor-joining trees. The scale bar represents 0.005 changes per nucleotide.

Supplementary Fig. S2 Two-dimensional thin-layer chromatography of polar lipids of *Microbispora clausenae* CLES2^T. Chloroform-methanol-water (65:25:4) was used in the first direction, followed by chloroform-acetic acid-methanol-water (40:7.5:6:2) in the second direction. Abbreviations: DPG; Diphosphatidylglycerol, PE; phosphatidylethanolamine, PI; phosphatidylinositol, PIDM; phosphatidylinositol dimannosides, PME; phosphatidylmethylethanolamine, UnGly; unknown glycolipid, Un Gly1; unknown glycolipid positive with ninhydrin and molybdenum blue spray, X; starting point.

Supplementary Fig. S3 Scanning electron micrograph of strain CLES2^T grown on ISP 3 for 14 days at 37 ^{o}C. Bar represents 5 μm.

Supplementary Figure S1

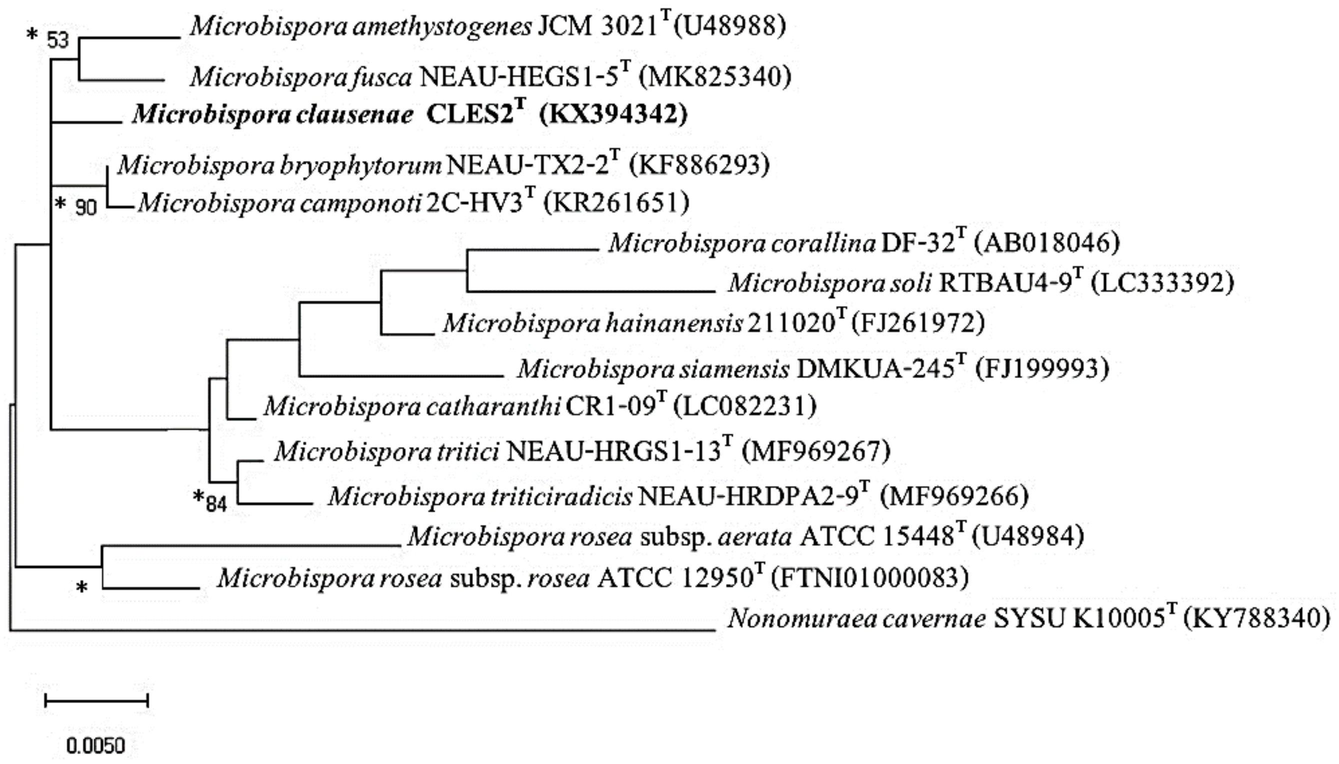

Supplementary Figure S2

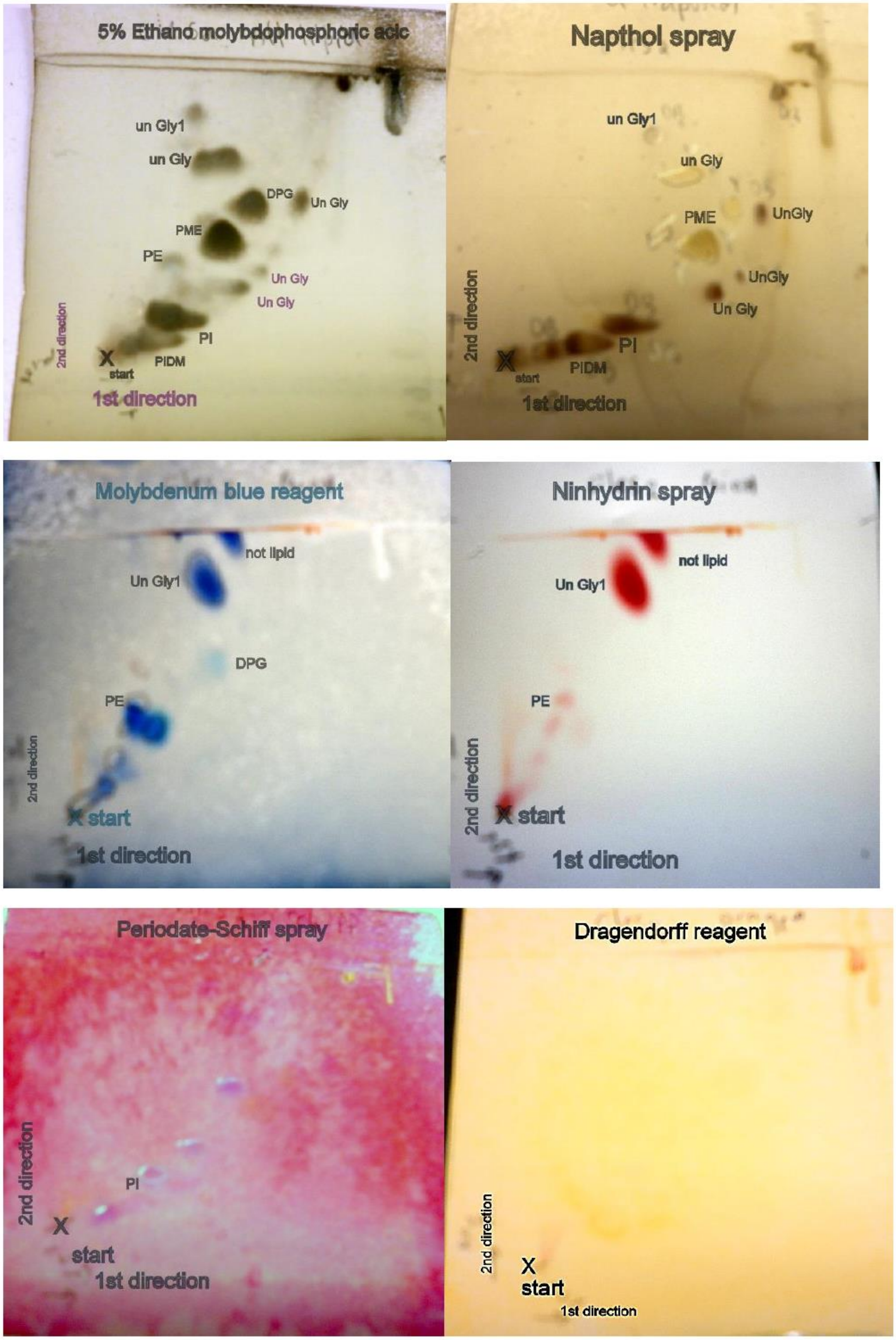

Supplementary Figure S3

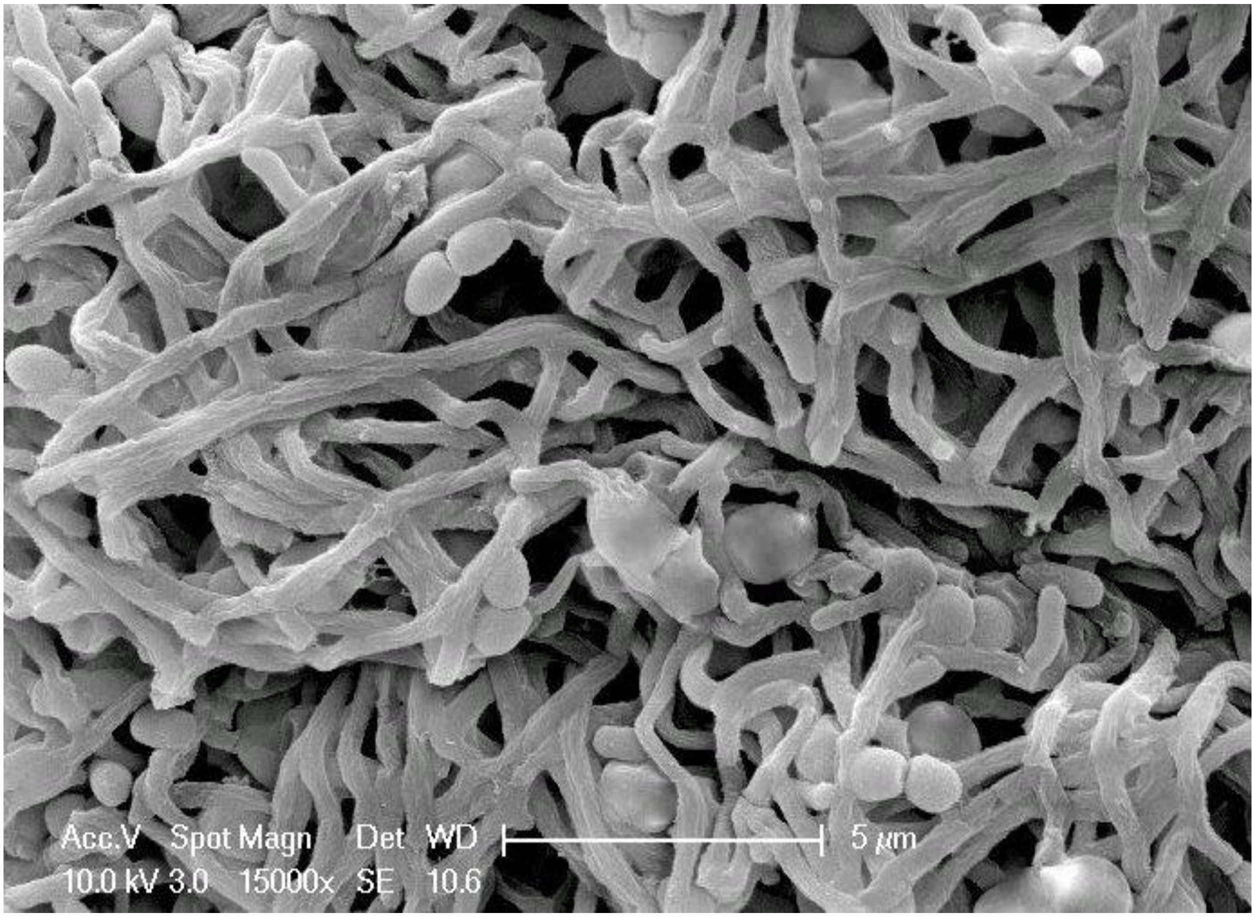

Supplementary Table S1. Morphology of *Microbispora clausenae* CLES2^{T}. Culture colour based on Kornerup and Wanscher [40].

Medium	Growth	Aerial mycelium (Colour)	Substrate mycelium (Colour)
ISP 2	Good	Reddish pink	Dark brown, dark brown pigment
ISP 3	Good	pink	Reddish pink
ISP 4	Poor	White	White
ISP 5	Poor	White	White
ISP 7	Poor	Reddish pink	Light brown, no melanin pigment
Bennett's agar	Good	Reddish pink	Reddish brown
Half strength potato dextrose agar	Good	Pale pink	Reddish brown
Nutrient agar	moderate	Not detect	Reddish brown
